# The relationship between adenoid hypertrophy and gastroesophageal reflux disease: A meta-analysis

**DOI:** 10.1097/MD.0000000000012540

**Published:** 2018-10-12

**Authors:** Xun Niu, Zeng-Hong Wu, Xi-Yue Xiao, Xiong Chen

**Affiliations:** Department of Obstetrics and Gynecology, Union Hospital, Tongji Medical College, Huazhong University of Science and Technology, Wuhan, China.

**Keywords:** adenoid hypertrophy, gastroesophageal reflux disease, meta-analysis

## Abstract

**Background:**

Gastroesophageal reflux disease (GERD) is believed to be associated with various manifestations in the otorhinolaryngology and has been found to be an additional risk factor for adenoid hypertrophy, but the causal relation between them is under controversial. We thus performed a meta-analysis to grade the strength of evidence and systematically explore whether adenoid hypertrophy correlates with GERD in the literature.

**Methods:**

A systematic literature search was performed using Medline via PubMed, Embase, CNKI, and Web of Science. Studies reporting the adenoid hypertrophy and GERD were identified for inclusion.

**Results:**

There were 6 studies that matched the selection criteria, and the total sample size of these studies was 548 cases. We identified a significant relationship between adenoid hypertrophy and GERD, with a pooled odds ratio of 4.12 (95% confidence interval [CI]: 1.32–12.93; *P* < .001). The results was significant in 24-hour pH monitoring subgroup analysis, with a corresponding value of 8.62 (95% CI: 4.06–18.27, *P* > .05) under the fixed-effects model. And the results was significant in *Helicobacter pylori* subgroup analysis, with a corresponding value of 2.39 (95% CI: 0.39–14.55, *P* < .05) under the random-effects model. Begg tests (*P* = .73) and Egger tests (*P* = .76) showed there were no obvious evidence to support publication bias in our study.

**Conclusion:**

This meta-analysis provided a strong correlation between adenoid hypertrophy and GERD, the children with adenoid hypertrophy had a higher incidence of GERD than healthy children, but the pathogenesis of GERD in adenoid hypertrophy awaits more investigations and suggests that we should not overlook GERD in clinical practice and an appropriate evaluation for GERD may be needed.

## 1 Introduction

Adenoid hypertrophy is one of the most common causes of pediatric upper airway infection, nasal obstruction, otitis media with effusion, and obstructive sleep apnea (OSA) syndrome, with adenoidectomy continuing as one of the commonest surgical procedures in clinical.^[1]^ Adenoid hypertrophy is believed a multifactorial process, recurrent acute viral, chronic bacterial infection, second-hand smoke exposure, allergic episodes can result in adenoid hyperplasia.^[2,3]^ Gastroesophageal reflux disease (GERD) is believed to be associated with various manifestations in the otorhinolaryngology (chronic rhinosinusitis, chronic cough, reflex apnea, asthma, recurrent otitis media, and chronic otitis media with effusion) and has been found to be an additional risk factor for adenoid hypertrophy.^[4]^ Refluxed materials of the stomach can reach the middle ear cleft probably due to premature Eustachian tube and the gastric contents can initiate an inflammatory cascade in the adenoid. It should be borne in mind that the typical symptoms or signs of GERD in patients with adenoid hyperplasia are questioned because GERD in patients with typical symptoms is significantly higher than in patients without symptoms.

Recently, more researches had looked at the relationship between adenoid hypertrophy and GERD in children, but the causal relation between them is under controversial. Carr et al^[5]^ studied the incidence of GERD in children under 2 years of age undergoing adenoidectomy and found that 88% of children aged 1 year or younger had GERD and 32% older than 1 year had GERD diagnosed. Harris et al^[6]^ first reported pepsin in the adenoid of children who were undergoing adenoidectomy but pepsin was not detected in any study or control adenoid. Other studies developed methods for *Helicobacter pylori* (Hp) detection in adenoid tissue and try to search for a correlation with GERD, and even studies were pay attention to antireflux treatment in the adenoid hypertrophy patients. Furthermore, more studies using endoscopy or 24-hour pH monitoring to document the acid reflux and the prevalence of GERD in patients with adenoid hypertrophy. But, there is also a controversy between adenoid hypertrophy and GERD because of the lake of enough studies.

We thus performed a meta-analysis to grade the strength of evidence and systematically explore whether adenoid hypertrophy correlates with GERD in the literature (supporting information: PRISMA Checklist).^[7]^

## 2 Materials and methods

### 2.1 Search strategy

Studies reporting the adenoid hypertrophy and GERD were identified for inclusion. To identify eligible original articles, we searched computerized databases, including Medline via PubMed, Embase, CNKI, and Web of Science using the following key terms: “adenoid,” “gastroesophageal reflux,” “reflux,” “adenoid hypertrophy,” “adenoidectomy” separated by the Boolean operator AND or OR. Articles were searched in the computerized databases up to March 2018, without limits of language. The computerized databases search was supplemented by a manual search of the bibliographies of all the retrieved articles. We screened the titles and abstracts of the identified studies, articles that could contain data regarding gastroesophageal reflux and adenoid hypertrophy were evaluated the full article. Two authors (ZHW and XN) independently searched for papers and screened the reference lists of retrieved papers to further identify potentially relevant publications. Discrepancies were resolved by consensus.

### 2.2 Inclusion and exclusion criteria and data extraction

Original studies were carefully checked. There were no country restrictions. The inclusion criteria were: all patients in the study had documented adenoid hypertrophy; patients were no previous history of adenoidectomy surgery, no history or treatment for GERD, and no ongoing acute infections; studies clearly defined the study and control groups and the group members, observational data were available; studies reported adenoid hypertrophy patients with pepsin concentrations, Hp detection, 24-hour pH monitoring to document the acid reflux or antireflux treatment. Case reports, non-English, abstracts, comments, review articles, duplicate publications, and editorials were excluded.

### 2.3 Data extraction

Information was collected for each publication concerning the author's name, publication year, study design, age, method of reflux evaluation, Hp/pepsin analysis method, study, and control group criteria.

### 2.4 Risk of bias and statistical analysis

We used the PRISMA statement^[8]^ to assess individual study quality and the risk of bias. Meta-analysis was performed using Cochrane statistical software Review Manager 5.3. Random-effects model was applied, depending on the *P*-value of the Chi-squared statistic when *P* was <.05 and Higgins *I*^2^ test were used to assess the heterogeneity (*I*^2^ < 25%, no heterogeneity; *I*^2^ = 25–50%, low heterogeneity; *I*^2^ = 50–75%, moderate heterogeneity; *I*^2^ > 75%, high heterogeneity). If *I*^2^-value >50%, the random effects model was used to combine effect size, and if *I*^2^-value <50%, the fix effects model was used to combine effect size. We also sought to perform subgroup analysis to determine the sources of heterogeneity. The pooled odds ratios (ORs) of different studies and corresponding 95% confidence intervals (CIs) were used to estimate the relationship between adenoid hypertrophy and GERD. The sensitivity analysis was repeated to assess the effects of single study on pooled estimates by removing individual study. The statistical significance was set at a *P* < .05.

## 3 Results

### 3.1 Study selection

**Figure 1:**
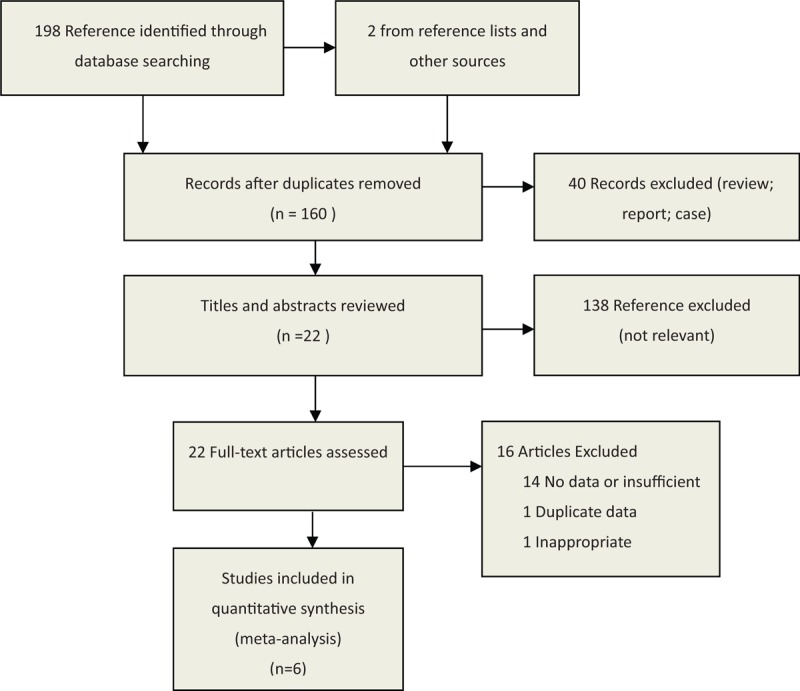
Search strategy to identify articles on the relationship between adenoid hypertrophy and gastroesophageal reflux.

Our search strategy identified 198 potentially relevant articles from electronic databases and 2 from reference lists and other sources. After excluding duplicates, 160 records remained. Reading the titles and abstracts of these 160 references led us to exclude 138 articles that did not meet the inclusion criterion. After reading the full text of the remaining 22 articles as possibly reporting the relationship between adenoid hypertrophy and GERD, 14 were excluded because they did not report sufficient data; 1 study was excluded because the outcome measure is inappropriate; and 1 study was duplicate. Ultimately, 6 eligible articles were identified.^[5,6,9–12]^ The selection process is shown in Figure 1 and the detailed information of each study is listed in Table 1.

**Table 1:**
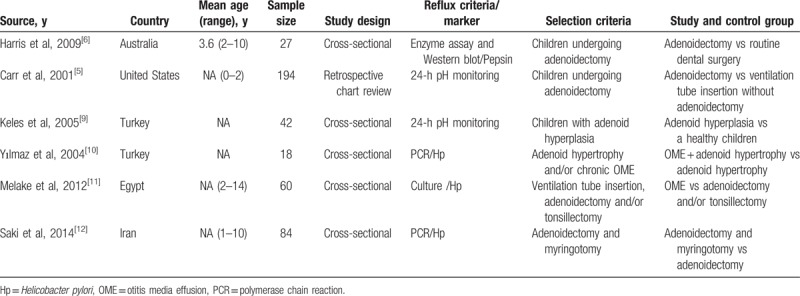
Description of included studies.

The meta-analysis consisted with a total sample size of 548. One study used immunohistochemistry or Western blot to detect the pepsin and 3 articles to detect the Hp in the adenoid specimen. Two articles used 24-hour pH monitoring to evaluate the relationship between adenoid hypertrophy and GERD in cases and controls.

### 3.2 Meta-analysis results

**Figure 2:**
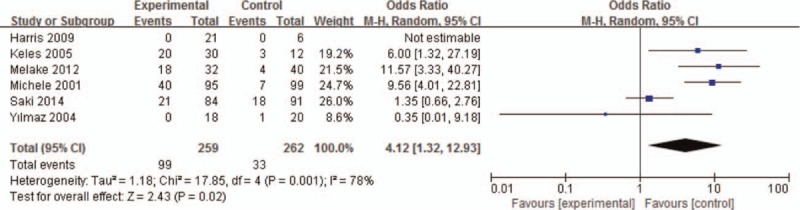
Relationship of adenoid hypertrophy and gastroesophageal reflux disease (GERD). The results indicated that adenoid hypertrophy was significantly associated with risk of GERD (odds ratio = 4.12, 95% confidence interval = 1.32–12.93).

**Figure 3:**
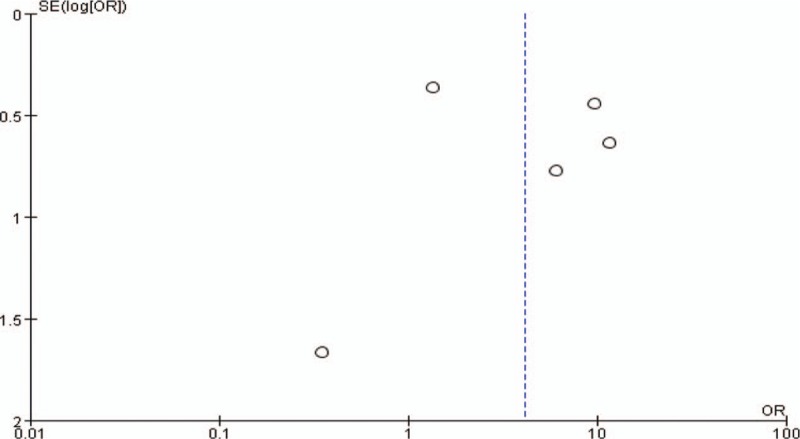
The funnel plot between relationship of adenoid hypertrophy and gastroesophageal reflux disease.

The forest plot result for association of adenoid hypertrophy with GERD is shown in Figure 2 and the funnel plot result is shown in Figure 3. We identified a significant relationship between adenoid hypertrophy and GERD, with a pooled OR of 4.12 (95% CI: 1.32–12.93; *P* < .001). The pooled data were calculated with the random-effects model as a high significant heterogeneity was found among the studies.

### 3.3 Subgroup analysis

#### 3.3.1 24-hour pH monitoring

**Figure 4:**
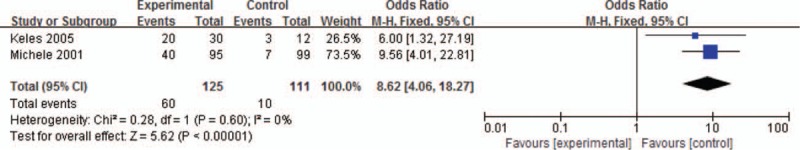
The forest plot of 24-hour pH monitoring subgroup analysis.

The results were significant in 24-hour pH monitoring subgroup analysis, with a corresponding value of 8.62 (95% CI: 4.06–18.27, *P* > .05) under the fixed-effects model. The forest plot about the subgroup analysis is showed in Figure 4. The results showed that the gastroesophageal reflux materials may play an important role in adenoid hypertrophy.

#### 3.3.2 Helicobacter pylori

**Figure 5:**
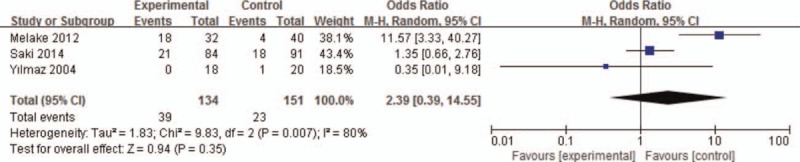
The forest plot of *Helicobacter pylori* subgroup analysis.

The results were significant in Hp subgroup analysis, with a corresponding value of 2.39 (95% CI: 0.39–14.55, *P* < .05) under the random-effects model. The forest plot about the subgroup analysis is showed in Figure 5. The results revealed that the Hp may be participating in adenoid hypertrophy pathogenesis.

### 3.4 Sensitivity analysis

The coupled forest plots show high heterogeneity (*I*^2^ = 78%) and when we removed Saki's study, the results dramatically influenced the pooled results (*I*^2^ decreased from 78% to 28%) in the meta-analysis under the random-effects model. The results of sensitivity analysis showed that the pooled ORs ranged from 3.09 (95% CI: 0.82–11.62) to 7.53 (95% CI: 3.33–17.00). Moreover, in subgroup analyses (*I*^2^ = 80% for Hp) showed high heterogeneity under the random-effects model and in 24-hour pH monitoring subgroup analysis showed no heterogeneity (*I*^2^ = 0%) under the fixed-effects model. However, in Hp subgroup, the results dramatically influenced by remove Melake study (*I*^2^ decreased from 80% to 0%).

### 3.5 Publication bias

**Figure 6:**
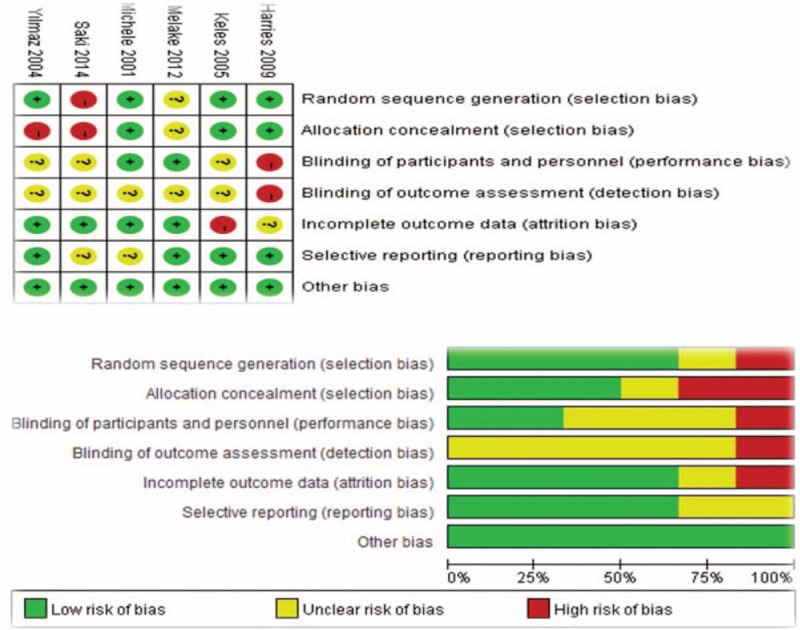
Risk of bias summary and graph: review authors’ judgments about each risk of bias item for each included study.

We also performed Egger and Begg funnel plots to assess the publication bias of the included studies and the results show that there is no publication bias. In addition, the trim and fill method showed that no study needed to be statistically corrected for funnel plot asymmetry and the methodologic quality of each included study is shown in Figure 6.

## 4 Discussion

This meta-analysis suggested that adenoid hypertrophy was significantly associated with increased risk of GERD (OR = 4.12; 95% CI: 1.32–12.93; *P* < .001) and the meta-analysis results were credible as there were no obvious bias as well as subgroups analyses. Begg tests (*P* = .73) and Egger tests (*P* = .76) showed there were no obvious evidence to support publication bias in our study. Although, the value of *I*^2^ = 78% (*I*^2^ > 50, *P* < .001), indicating that there existed high heterogeneity among the studies. But we performed the subgroup analysis to determine the sources of heterogeneity so the results of our study could represent the true relationship between adenoid hypertrophy and GERD. Meanwhile, sensitivity analysis exhibited that after any single study was omitted the overall results and conclusion still held. Therefore, we have confidence to believe that a strong association between adenoid hypertrophy and GERD.

The role of GERD in adenoid hypertrophy pathogenesis is unclear, with some studies reporting Hp in patients with adenoid hypertrophy suffering from GERD and other reports using 24-hour pH monitoring to find the associated with GERD. Harris et al first studied pepsin proteins and genes in hypertrophic adenoids, but the pepsin was not detected in any study or control adenoid group, so more studies are needed to convince it. The standard diagnostic of GERD is 24-hour dual-probe esophageal pH monitoring, it can detect episodes of reflux not only laryngopharyngeal but also gastroesophageal reflux. But in our meta-analysis, we only contain the data from gastroesophageal reflux. A representative study was Phipps's research and he found that the frequency of GERD was 64.5%.^[13]^ These results supported that GERD may play an important role in the etiology of adenoid hyperplasia. Our findings and his conclusions are consistent, the summary OR was 8.62 (95% CI: 4.06–18.27) for 24-hour pH monitoring subgroup analysis in our meta-analysis under the fixed-effects model.

Recently, one increasing interest in some studies was to compare Hp prevalence rates in the adenoid hypertrophy of pediatric patients. Hp is a gram-negative bacterium and the etiologic agent of some extra gastrointestinal diseases, and the condition of the nasopharynx is more favorable for the growth of this bacterium, especially in adenoid hypertrophy.^[14,15]^ We included 3 articles in our meta-analysis for the detection of Hp in the adenoid specimen and in Hp subgroup analysis, with a corresponding value of 2.39 (95% CI: 0.39–14.55, *P* < .05) under the random-effects model. Thus, we can postulate that the Hp may be participating in adenoid hypertrophy pathogenesis. Another increasing interest is some studies focus on the antireflux therapy and trying to establish a relationship between them. Iqbal et al^[16]^ try to establish the efficacy of proton pump inhibitors (PPIs) in the treatment of adenoid hypertrophy in children but his study does not demonstrate any efficacy of PPIs for adenoid hypertrophy in children. His study may present bias, as he observed the adenoid size in the treatment group and control group so there must be more evidence verifying that the antireflux therapy is useful. Stapleton and Brodsky^[17]^ reported a case of an infant whose adenoid hypertrophy resolved after PPI therapy, which may support the idea of GERD causing the adenoid hypertrophy. While in our study, no antireflux therapy articles meet our inclusion criteria.

From this meta-analysis, we may arrive at a conclusion that the GERD participation in adenoid hypertrophy pathogenesis. On one hand, for patients with adenoid hypertrophy and GERD, we can try antireflux treatment (such as PPIs) first, if the adenoid size did not decreased or smaller changes then may be adenoidectomy surgical procedure is needed. On the other hand, if we suspected patients with adenoid hypertrophy with GERD signs and symptoms (such as frequent throat clearing, sore throat and posterior laryngitis with hoarseness, chronic cough and even laryngeal, and subglottic stenosis^[18]^) endoscopic assessment or 24-hour dual-probe esophageal pH monitoring should be performed. Thus, we can identify high-risk patients and treated GERD to lower unnecessary surgical procedure. But many of parents especially of healthy children or smaller children may be did not accept this application, as is an invasive method.

This is the first meta-analysis to approve the association of adenoid hypertrophy with GERD. But some potential limitations of the meta-analysis should be taken into consideration when interpreting the results of our study. Firstly, the sample size was relatively not enough and may affect the accuracy of our results and much large-scale studies should be performed to convince it. Secondly, although we explored the source of heterogeneity by subgroup analysis in our study, we could not explore the main heterogeneity. On the contrary, we could not do the meta-regression because of the few studies. Thirdly, the results may also be biased by different measurement techniques to detect Hp (polymerase chain reaction/culture). So our results should be interpreted with caution and need further researches. Despite there are limitations, our analysis in terms of showing a strong and clear association between adenoid hypertrophy and GERD.

## 5 Conclusion

This meta-analysis provided a strong correlation between adenoid hypertrophy and GERD. The children with adenoid hypertrophy had a higher incidence of GERD than healthy children, but the pathogenesis of GERD in adenoid hypertrophy awaits more investigations and suggests that we should not overlook GERD in clinical practice and an appropriate evaluation for GERD may be needed.

## Author contributions

NX and WZH contributed equally to the work as co-first authors. NX and WZH designed the study, analyzed the data, and wrote the manuscript; XXY and CX edited the manuscript as the corresponding author. In addition, all authors approved the final draft.

**Software:** Xi-Yue Xiao.

**Visualization:** Xiong Chen.

**Writing – original draft:** Zeng-Hong Wu.

**Writing – review & editing:** Zeng-Hong Wu, Xun Niu.

## References

[1] Shatz A Indications and outcomes of adenoidectomy in infancy. Ann Otol Rhinol Laryngol 2004;113:835–8. 15535148 10.1177/000348940411301011

[2] Robb PJ Adenoidectomy: does it work? J Laryngol Otol 2007;121:209–14. 16719956 10.1017/S0022215106001563

[3] Sataloff RT Scott-Brown's otolaryngology. JAMA 1989;262:2614–5.

[4] Matthew Crapko BA Kerschner JE Michael Syring BA Role of extra-esophageal reflux in chronic otitis media with effusion. Laryngoscope 2007;117:1419–23. 17585281 10.1097/MLG.0b013e318064f177

[5] Carr MM Poje CP Ehrig D Incidence of reflux in young children undergoing adenoidectomy. Laryngoscope 2001;111:2170–2. 11802019 10.1097/00005537-200112000-00018

[6] Harris PK Hussey DJ Watson DI Reflux changes in adenoidal hyperplasia: a controlled prospective study to investigate its aetiology. Clin Otolaryngol 2009;34:120–6. 19413609 10.1111/j.1749-4486.2008.01852.x

[7] Stewart LA Clarke M Rovers M Preferred reporting items for systematic review and meta-analyses of individual participant data: the PRISMA-IPD statement. JAMA 2015;313:1657–65. 25919529 10.1001/jama.2015.3656

[8] Moher D Liberati A Tetzlaff J Preferred reporting items for systematic reviews and meta-analyses: the PRISMA statement. Revista Española De Nutrición Humana Y Dietética 2014;18:e123. PMC3090117 21603045

[9] Keles B Ozturk K Arbag H Frequency of pharyngeal reflux in children with adenoid hyperplasia. Int J Pediatr Otorhinolaryngol 2005;69:1103–7. 16005352 10.1016/j.ijporl.2005.02.019

[10] Yilmaz MD Aktepe O Çetinkol Y Does *Helicobacter pylori* have role in development of otitis media with effusion? Int J Pediatr Otorhinolaryngol 2005;69:745–9. 15885326 10.1016/j.ijporl.2004.12.009

[11] Melake NA Shaker GH Salama MA Incidence of *Helicobacter pylori* infection and their clarithromycin-resistant strains in otitis media with effusion regarding phenotypic and genotypic studies. Saudi Pharm J 2012;20:345–53. 23960809 10.1016/j.jsps.2012.02.004 PMC3745192

[12] Saki N Zadeh ARS Jonaky RS The prevalence rate of *Helicobacter pylori* infection in, chronic otitis media with effusion patients. Jundishapur J Microbiol 2014;7:e15694. 25147680 10.5812/jjm.15694 PMC4138655

[13] Phipps CD Wood WE Gibson WS Gastroesophageal reflux contributing to chronic sinus disease in children: a prospective analysis. Arch Otolaryngol Head Neck Surg 2000;126:831–6. 10888994 10.1001/archotol.126.7.831

[14] Kowalski M *Helicobacter pylori* (*H. pylori*) infection in coronary artery disease: influence of *H. pylori* eradication on coronary artery lumen after percutaneous transluminal coronary angioplasty. The detection of *H. pylori* specific DNA in human coronary atheroscleroti. J Physiol Pharmacol 2001;52:3–1. 11795863

[15] Farhadi M Noorbakhsh S Tabatabaei A Searching the *H. pylori*; serology & PCR in children with adenoid hypertrophy and rhino sinusitis: a cross sectional study, Tehran, Iran. Med J Islamic Repub Iran 2013;27:77–82. PMC3610311 23741169

[16] Iqbal FR Goh BS Mazita A The role of proton pump inhibitors in adenoid hypertrophy in children. Otolaryngol Head Neck Surg 2012;147:329–34. 22496101 10.1177/0194599812444528

[17] Stapleton A Brodsky L Extra-esophageal acid reflux induced adenotonsillar hyperplasia: case report and literature review. Int J Pediatr Otorhinolaryngol 2008;72:409–13. 18160139 10.1016/j.ijporl.2007.11.003

[18] Koufman JA The otolaryngologic manifestations of gastroesophageal reflux disease (GERD): a clinical investigation of 225 patients using ambulatory 24-hour pH monitoring and an experimental investigation of the role of acid and pepsin in the development of laryngeal. Laryngoscope 1991;101:1–78. 10.1002/lary.1991.101.s53.1 1895864

